# Personality disorders, violence and antisocial behaviour: updated systematic review and meta-regression analysis

**DOI:** 10.1192/bjp.2024.226

**Published:** 2025-07

**Authors:** Rachel T.S. Chow, Rongqin Yu, John R. Geddes, Seena Fazel

**Affiliations:** Department of Psychiatry, University of Oxford, Warneford Hospital, Oxford, UK; Oxford Health NHS Foundation Trust, Warneford Hospital, Oxford, UK; NIHR Oxford Health Biomedical Research Centre, Warneford Hospital, Oxford, UK

**Keywords:** Personality disorders, forensic psychiatry, meta-analysis, systematic review, observational study

## Abstract

**Background:**

Links between personality disorders and antisocial outcomes has not examined individual personality disorders, and the contribution of comorbidities remain uncertain. Previous systematic reviews are dated.

**Aims:**

To synthesise evidence from observational studies on the risk of antisocial outcomes and recidivism associated with personality disorders.

**Method:**

We searched six bibliographic databases (up to March 2024) for observational studies examining the risk of antisocial behaviour, interpersonal violence and recidivism in individuals diagnosed with personality disorders, compared to controls. We explored sources of heterogeneity using subgroup analyses and meta-regression.

**Results:**

We identified 21 studies involving 83 418 individuals with personality disorders from 10 countries examining antisocial and violent outcomes (Aim 1), and 39 studies of 14 131 individuals from 13 countries with recidivism (or repeat offending) as the outcome (Aim 2). We found increased risks of violence among individuals with any personality disorder (odds ratio 4.5, 95% CI 3.0–6.7), particularly antisocial personality disorder (odds ratio 7.6, 95% CI 5.1–11.5) and borderline personality disorder (odds ratio 2.6, 95% CI 1.8–3.9). Individuals with any personality disorder (odds ratio 2.3, 95% CI 2.0–2.6) and antisocial personality disorder (odds ratio 2.8, 95% CI 1.6–4.9) also demonstrated an elevated risk of recidivism. Personality disorder types and comorbid substance use disorder were associated with between-study heterogeneity.

**Conclusions:**

The assessment and management of personality disorders should be considered as part of violence prevention strategies. Improving identification and treatment of comorbid substance misuse may reduce adverse outcomes in individuals with personality disorders.

The global prevalence of personality disorders in community settings is approximately 8%.^1^ Personality disorders are associated with a range of adverse outcomes, including suicidality, substance misuse and physical and psychiatric comorbidities.^2–4^ A previous meta-analysis of 14 primary studies reported a threefold increased risk of antisocial behaviour and interpersonal violence perpetration in individuals with personality disorders compared with the general population.^5^ However, this review included studies reported up to 2009, and since then many new investigations have been published.^6,7^ Moreover, the previous review reported high between-study heterogeneity but did not find explanations for this, apart from higher odds in people with antisocial personality disorder (ASPD). This was mainly because of the limited number of primary studies. Notably, the risk of violence in other personality disorders remained unclear. The link between individual personality disorders and antisocial outcomes may vary because of their clinical characteristics and varying comorbidity patterns. For instance, impulsivity, a transdiagnostic feature of both ASPD and borderline personality disorder (BPD), has been associated with physical aggression and recidivism.^8,9^ BPD is also common in forensic mental health settings, with prevalence estimates ranging from 20% to 30%.^10–12^ Other features, such as mood instability, paranoid ideation, obsessionality and suicidality, occur in individual personality disorders, and may be associated with specific outcomes.

## Aims of the review

We report an updated systematic review and meta-analysis of observational studies examining the risks of antisocial behaviour (Aim 1) and recidivism (Aim 2) in individuals with personality disorders compared to control groups without personality disorders. This could inform risk assessment and management in different personality disorders, service provision and identify priorities for future research.

## Method

We conducted this meta-analysis following the Preferred Reporting Items for Systematic Reviews and Meta-Analyses (PRISMA) guidelines (Supplementary Appendix A available at https://doi.org/10.1192/bjp.2024.226).^13^ The review protocol is registered on the PROSPERO database (CRD42021247237). We identified observational studies (in published and grey literature) reporting the risk estimates of antisocial behaviour and recidivism in individuals diagnosed with personality disorders released between 1 January 1966 and 14 March 2024. This review adopted the methodology of the systematic review conducted by authors R.Y., J.R.G. and S.F. for the period between 1966 and 2009.^5^ We conducted an updated literature search (from 1 January 2009 to 14 March 2024) in databases including Medline, Embase, PsycInfo, CINAHL, US National Criminal Justice Reference System (NCJRS) and Web of Science. We used the same search strategy as the previous systematic review, which comprised a combination of search terms for personality disorders (i.e. personality disorder*, personality pathology, axis II, personality dysfunction, personality abnormality and abnormal personality) and antisocial behaviour (i.e. viol*, offen*, aggress*, assault*, antisocial, anti-social, dangerous*, crim*, delinquen* and unlawful*) and recidivism (i.e. recidi*, reoffend*, repeated offend*, rearrest, reconvict*, reincarcerat*, revoke* and recur*). Personality disorders are often investigated concurrently with other psychiatric disorders for violent outcomes, but information on personality disorders is often not mentioned in titles and abstracts in these studies. We therefore included more general psychiatric disorder-related terms (i.e. mental disorder*, mental illness* and psychiatric disorder*) to enhance search sensitivity. Non-English language articles were translated and examined for eligibility. Reference lists of included papers were scanned to further identify potentially eligible articles. We corresponded with authors when clarification and additional data were required. No informed consent from participants was required for this review as only secondary data from existing research were collected and analysed.

### Study eligibility

We included studies that met the following criteria: (a) with a cohort, case–control or cross-sectional design; (b) reporting on individuals diagnosed with personality disorders, defined according to validated diagnostic criteria using clinical and/or (semi-)structured interviews; (c) reporting the risk of antisocial behaviour in individuals with personality disorders compared to those without personality disorders in the general population (Aim 1) or the risk of reoffending/recidivism in individuals with a history of criminal behaviour with personality disorders compared to individuals with a history of criminal behaviour but without personality disorders (Aim 2); and (d) reporting the risk of antisocial behaviour and/or reoffending in terms of study-level quantitative data, which allows the calculation of odds ratios. Studies that reported a specific type of antisocial behaviour (e.g. intimate partner violence, sexual assault) and provided no appropriate comparison data were excluded. One of the authors (R.T.S.C.) conducted the initial screening, identified full texts and selected studies for inclusion. In addition, an independent reviewer, Phoebe Homer, independently selected studies for inclusion from a randomly sampled 20% of the identified full texts. Any discrepancies between R.T.S.C. and P.H. were discussed with a third author (R.Y. or S.F.) until consensus was achieved. One study was excluded as the study sample was limited to individuals with available data on functional impairment, rather than violent outcomes.^14^ When multiple papers on the same dataset were retrieved, we included the paper reporting the most complete dataset to avoid duplicated samples. In this meta-analysis, we excluded two studies with overlapping samples.^7,15^

### Data extraction

Data extraction began on 15 February 2022. Using a standardised extraction form, data and information on the following study characteristics were independently recorded by R.T.S.C. and P.H. for each study: publication year, study period, country, design, sample size, diagnostic criteria for personality disorder diagnosis, personality disorder diagnosis, method of outcome ascertainment, adjusted variables and participants’ demographic information (age and gender). Odds ratios with 95% confidence intervals were extracted or calculated from the number of participants with or without personality disorder cross-classified by antisocial or reoffending outcomes, either by direct extraction if reported or by derivation from summary statistics and prevalence data. Risk estimates with and without adjustments were extracted if both were reported. We corresponded with primary study authors to resolve uncertainties about extracted data. For interrater reliability in effect sizes, Spearman's correlation coefficient was 0.999, indicating almost perfect agreement between data extractors. There were only four disagreements in the extracted raw data for effect size and 95% confidence interval calculations, which were discussed between extractors and consensus reached.

### Data analysis

We conducted meta-analyses on extracted odds ratios and corresponding 95% confidence intervals. Fixed-effects models were used when heterogeneity was considered low to moderate, as indicated by the *I*^2^ statistic (see below for details). Random-effects models, which assumes variance in the effect estimates between the included studies given their varying sizes, designs and sample characteristics, were used when heterogeneity was high. Random-effects models account for the high overall between-study heterogeneity by assigning similar weights to each study in the meta-analysis, while fixed-effects models assign more weights to larger studies assuming all studies have identical true effect sizes.^16^ When both adjusted and unadjusted risk estimates were reported for a single association, the adjusted one was used for the main meta-analysis. We performed sensitivity analyses on studies examining any criminality (including violence) as an outcome.

Heterogeneity was assessed using the *I*^2^ statistic, which estimates the observed dispersion attributable to variation rather than chance across the pooled studies in a meta-analysis. The *I*^2^ statistic is expressed as a percentage, with the following recommended thresholds: low (0–40%), moderate (30–60%), substantial (50–90%) and considerable (75–100%).^17^ We explored sources of heterogeneity using subgroup and meta-regression analyses on a series of pre-determined study characteristics, including publication year, geographical region, study design, adjustment, comparison group, diagnosis, diagnostic criteria, average age, sample size, and ascertainment of outcomes. Subgroup analyses were carried out using non-overlapping data. In meta-regression, categorical independent variables were entered individually and then in multivariable models. To measure the incidence of violence, antisocial behaviour and recidivism attributable to personality disorders, we calculated the population attributable risk fraction by dividing the difference between the base rate *r* (i.e. the number of individuals involved in criminal behaviour per 1000 individuals with personality disorders) and *r*_0_ (i.e. the number of individuals involved in criminal behaviour per 1000 controls without personality disorders) by the rate among individuals with personality disorders (*r*). We investigated publication bias using Egger's test (i.e. weighted regression method).^18^ We also performed leave-one-out sensitivity analyses to assess the influence of outliers on the overall risk estimates. All statistical analyses were conducted on STATA-MP, version 17.0 for MacOS, using the *metan*, *metareg*, *metabias* and *metainf* commands.

### Quality assessments

The risk of bias and methodological quality of each included study was assessed independently by two researchers (R.T.S.C. and independent reviewer Phoebe Homer) using the Newcastle–Ottawa scale (NOS).^19^ Interrater reliability was calculated with a two-way random-effects intraclass correlation coefficient,^20^ which was 0.86, indicating excellent agreement.^21^ Study quality was assessed in terms of sample selection, comparability between individuals with and without personality disorders, ascertainment of antisocial behaviour and recidivism outcomes and the rigour of statistical analyses. For studies with a case–control or cohort design, the maximum score was 9. For cross-sectional studies, we used an adapted version of the NOS,^22^ with a potential total score ranging from 0 to 8.

## Results

**Fig. 1:**
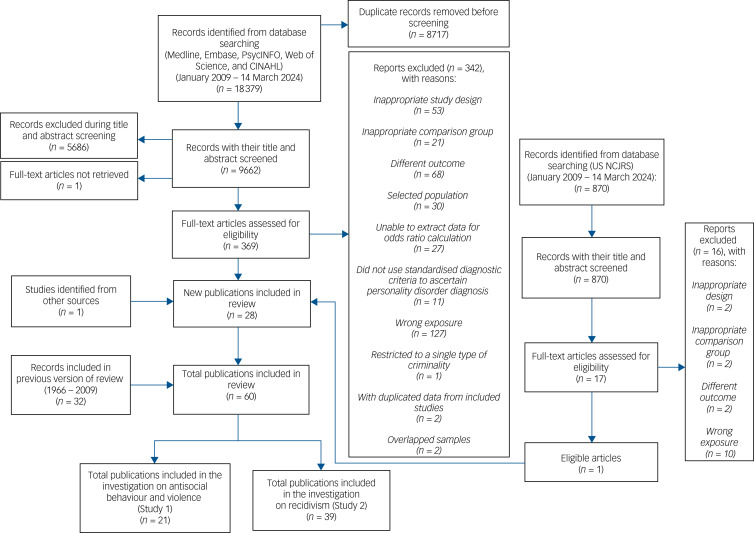
Flowchart outlining the search strategy. NCJRS, National Criminal Justice Reference System.

The updated systematic search yielded 9662 unique records, of which we screened 369 full-text articles for eligibility (Fig. 1). We identified 60 publications that reported on 71 separate relevant outcomes. The updated search included 28 new studies (with 32 reported outcomes) while the previous review included 32 studies (with 39 reported outcomes). Among the included cohort and case–control studies, 14 were considered high quality (scoring ≥7 on the NOS) while the remaining studies scored 6 or lower (*n* = 29). The median scores for cohort studies and case–control studies were both 6 (mean 6.1; interquartile range [IQR] 5–7 for cohort studies, mean 6; IQR 6 for case–control studies). Most cross-sectional studies scored 6 or lower, with a median score of 5 (mean 4.7; IQR 3–6).

### Study 1: risk of violence in personality disorder

There were 16 studies reporting outcomes on the links between personality disorders and violence in 76 647 individuals diagnosed with personality disorders (Supplementary Appendix Table B.1).^6,23–37^ Of individuals diagnosed with personality disorders, 7186 (9.4%) exhibited violent behaviour. These cases were compared with 6 441 949 individuals in the general population, of whom 127 191 (2.0%) perpetrated some form of violence.

Eligible studies provided data on all personality disorders (*k* = 6),^24,29–33^ ASPD (*k* = 7)^6,23,25,28,34,35,37^ and BPD (*k* = 3).^26,27,36^ One study also reported risk estimates in other personality disorders.^6^ Studies were from nine countries: three each from Denmark, the USA and the UK, two from Sweden and one each from Canada, China, Finland, Israel and the Netherlands. Three studies reported both antisocial behaviour and violent outcomes.^25,28,36^

#### Any personality disorders

**Fig. 2:**
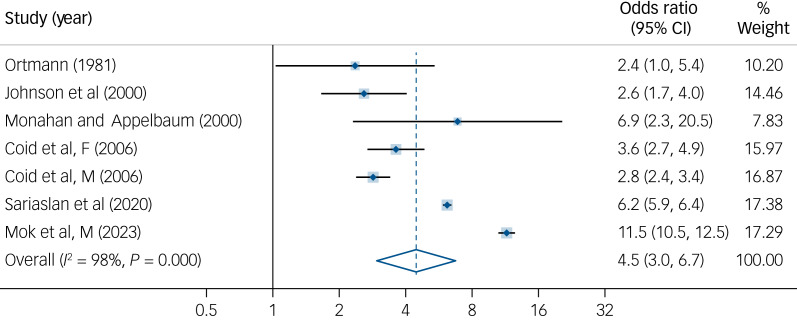
Risk estimate for violence in individuals diagnosed with all personality disorders compared to the general population.^24,29–33^ F, female sample; M, male sample.

There was an association between personality disorders and increased risk of violence (random-effects odds ratio 5.4, 95% CI 3.5–8.2) with considerable heterogeneity between studies (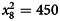, *I*^2^ = 98%, *P* < 0.001). The odds ratios ranged from 2.4 to 17.2. Leave-one-out sensitivity analyses revealed that the most influential outlier was Mok (2023F) with odds ratio 17.2 (95% CI 14.9–19.9).^30^ After exclusion, the increased risk of violence in personality disorders remained significant (odds ratio 4.5, 95% CI 3.0–6.7) with considerable heterogeneity (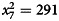, *I*^2^ = 98%, *P* < 0.001) (Fig. 2). When excluding low-quality studies, the odds ratio was 4.3 (95% CI 2.8–6.6). Although there were differences in the reported risk of violence between studies that included ASPD (or did not report the ASPD proportion; odds ratio 4.9, 95% CI 3.2–7.6) versus those investigations where ASPD was excluded (odds ratio 2.6, 95% CI 1.7–4.0), the latter risk estimate was significant. In addition, where ASPD proportions were small (three studies with 6.2%, 6.4% and 20.0%), there was increased violence risk (odds ratio ranged from 2.8 to 6.2).

#### Antisocial personality disorder

**Fig. 3:**
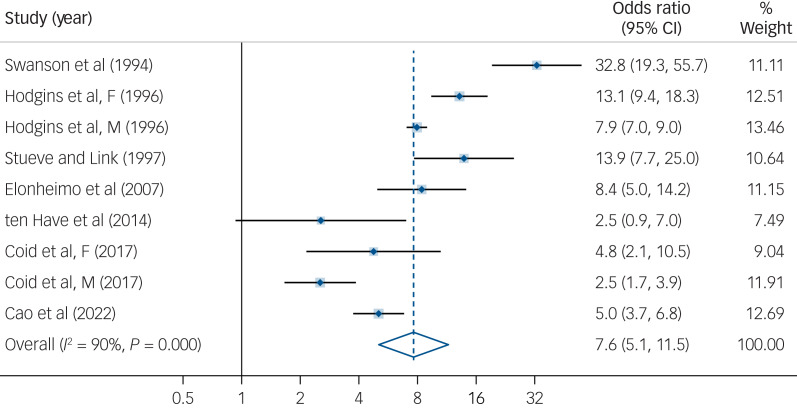
Risk estimate for violence in individuals diagnosed with antisocial personality disorder compared to the general population.^6^^,^^23^^,^^25^^,^^28^^,^^34,35^^,^^37^ F, female sample; M, male sample.

There was an increased risk of violence (odds ratio 7.6, 95% CI 5.1–11.5) associated with individuals diagnosed with ASPD compared to general population controls, with considerable between-study heterogeneity (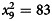, *I*^2^ = 90%, *P* < 0.001) (Fig. 3). Odds ratios ranged widely from 2.5 to 32.8. There was no significant difference between ASPD and all personality disorder samples in their associated risk of violence. The population attributable risk of violence associated with ASPD was 2 per 1000 individuals, with 13.0% of violent incidents attributable to ASPD.

#### Borderline personality disorder

There was an association between BPD and violence (odds ratio 2.6, 95% CI 1.8 to 3.9) with substantial heterogeneity (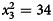, *I^2^* = 94%, *P* ≤ 0.001) with odds ratios ranging from 1.5 to 3.9 among the three included studies. The risk of violence associated with BPD was significantly lower than that in ASPD. The population attributable risk for violence is 1 per 1000 individuals diagnosed with BPD, with a population attributable risk fraction of 3.0%.

#### Other personality disorders

Among samples with both genders combined, from one study,^6^ there was association with violence in paranoid personality disorder (odds ratio 1.6, 95% CI 1.1–2.3). However, associations between violence and narcissistic personality disorder (odds ratio 2.6, 95% CI 1.0–6.8), histrionic personality disorder (odds ratio 1.7, 95% CI 0.8–3.9), schizoid personality disorder (odds ratio 1.3, 95% CI 1.0–1.7) and obsessive–compulsive personality disorder (odds ratio 1.3, 95% CI 0.9–1.8) were increased but did not reach statistical significance. In contrast, there were no clear associations between violent outcomes and avoidant personality disorder (odds ratio 0.8, 95% CI 0.5–1.2), dependent personality disorder (odds ratio 0.8, 95% CI 0.4–1.6) or schizotypal personality disorder (odds ratio 0.8, 95% CI 0.5–1.3).

#### Gender

There was no significant difference by gender in the risk of violence associated with any personality disorder (women: odds ratio 3.6, 95% CI 2.7–4.9; men: odds ratio 4.8, 95% CI 1.8–13.2) and ASPD (women: odds ratio 8.5, 95% CI 3.2–22.7; men: odds ratio 5.4, 95% CI 3.3–8.9).

#### Comorbidity with substance misuse

**Fig. 4:**
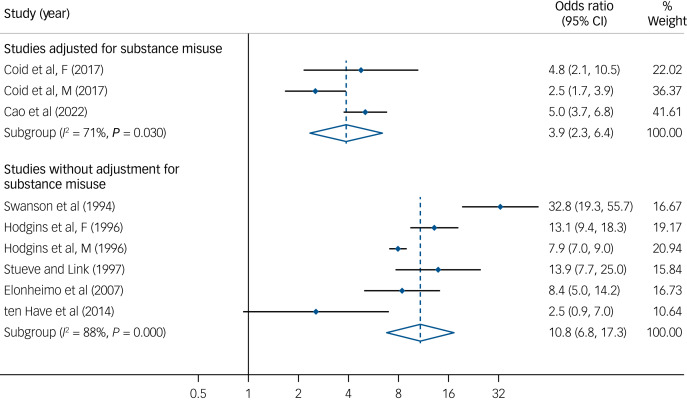
Risk estimates for violence in antisocial personality disorder with and without adjustment on substance use disorder.^6,23,25,28,34,37^

Among individuals with any personality disorder, the prevalence of substance use disorder (SUD) was 28.3% in men and 10.3% in women.^30^ In ASPD samples, the prevalence of substance misuse (i.e. alcohol misuse and drug use) ranged from 10.4% to 19.7%.^23^ The prevalence of substance misuse ranged from 7.2% to 46.1% in BPD samples.^26,36^ ASPD studies that adjusted for SUDs reported significantly smaller effect sizes for violence than studies without adjustment (odds ratio, 3.9 [2.3, 6.4] *v.* 10.8 [6.8, 17.3]) (Fig. 4).

One study provided data for the calculation of violence risk associated with any personality disorder with and without comorbid SUDs.^30^ The risk of violence associated with personality disorder–SUD comorbidity (odds ratio 29.9, 95% CI 13.2–68.0) was higher than personality disorder without SUD comorbidity (odds ratio 14.0, 95% CI 9.4–20.8) but confidence intervals overlapped owing to the small sample size.

#### Other characteristics

For studies investigating links with any personality disorder, we found differences in violence risk by study design: cohort studies reported significantly larger effect sizes than case–control studies and cross-sectional investigations (Table 1). No significant difference was found for ASPD studies. Subgroup analyses on BPD were not possible due to the limited number of primary studies.

**Table 1:** Risk estimates for violence in any personality disorder by study characteristics

Sample or study characteristics	Number of studies	Number with personality disorder (violent cases with personality disorder)	Random-effects odds ratio (95% CI)
Study period (*k* = 7)			
Studies conducted before 1998	1	135 (6)	2.4 (1.0–5.4)
Studies conducted in and after 1998	6	35 576 (3484)	4.8 (3.1–7.4)
Study region (*k* = 7)			
USA	2	123 (44)	3.7 (1.5–9.3)
Scandinavia	3	33 116 (2984)	6.5 (3.7–11.2)
The rest of the world	2	2472 (462)	3.1 (2.5–3.9)
Design (*k* = 7)			
Case–control	2	238 (45)	2.5 (1.7–3.8)
Cohort	3	33 001 (2983)	8.1 (4.7–14.2)
Cross-sectional	2	2472 (462)	3.1 (2.5–3.9)
Adjustment by sociodemographic and/or clinical variables (*k* = 7)			
With adjustment	2	2472 (462)	3.1 (2.5–3.9)
Without adjustment	5	33 239 (3028)	5.3 (3.4–8.4)
Adjustment by SUDs (*k* = 7)			
With adjustment	2	2472 (462)	3.1 (2.5–3.9)
Without adjustment	5	33 239 (3028)	5.3 (3.4–8.4)
Comparison group (*k* = 7)			
General population	4	3526 (718)	4.7 (1.7–13.1)
General population without psychiatric disorders	3	32 185 (2772)	4.0 (2.3–7.1)
Number of cases (*k* = 7)			
<1000 cases	3	258 (50)	3.0 (1.8–4.9)
>1000 cases	4	35 453 (3440)	5.3 (3.2–8.6)
Diagnostic criteria (*k* = 7)			
DSM criteria	4	2595 (506)	3.1 (2.5–3.8)
ICD criteria	3	33 116 (2984)	6.5 (3.7–11.2)
Data source (*k* = 7)			
Register	3	33 116 (2984)	6.5 (3.7–11.2)
Self-report	2	2472 (462)	3.1 (2.5–3.9)
Combination of registry and self-report sources	2	123 (44)	3.7 (1.5–9.3)

SUD, substance use disorder.

#### Meta-regression and publication bias

Meta-regression analyses found no study characteristic to be significantly associated with heterogeneity. Egger's test found no clear evidence of publication bias in studies reporting violent outcomes in all personality disorders (*t* = −0.90, *P* = 0.84), ASPD (*t* = −0.18, *P* = 0.93) and BPD (*t* = −5.39, *P* = 0.15).

#### Sensitivity analysis: any criminality

When investigating any antisocial behaviour (including violence) as the outcome, we found an increased risk in individuals diagnosed with any personality disorder, ASPD and BPD compared to general population controls, while an equivocal association was found in schizotypal personality disorder (Supplementary Appendix Table C.1; Appendix Figs C.1 and 2).^25,28,36,38–42^ There was moderate between-study heterogeneity in any personality disorder and considerable heterogeneity in ASPD and BPD studies.

In studies examining all personality disorders, studies with less than 100 personality disorder cases reported significant higher risk estimates than studies reporting over 1000 personality disorder cases (Supplementary Appendix Table C.2). No subgroup analysis was performed on individual personality disorder samples owing to an insufficient number of primary studies (*k* < 5). Egger's test found no significant evidence of publication bias in studies reporting any antisocial behaviour (including violence) associated with all personality disorders (*t* = 1.01, *P* = 0.39) and ASPD (*t* = −1.02, *P* = 0.42).

### Study 2: risk of repeat offending (recidivism) in personality disorders

We identified 39 studies reporting recidivism data on 14 131 individuals with a history of criminal behaviour diagnosed with at least one personality disorder (Supplementary Appendix Table B.2).^8,43–80^ Eighteen additional studies were included in this update.^8,45–47,49–51,54,57,59–61,63,66,67,72,73,80^ Of individuals with a history of criminal behaviour who had personality disorders, 6420 (45.4%) reoffended. These individuals were compared with 155 925 individuals with a history of criminal behaviour with or without psychiatric disorders, among whom 61 282 (39.3%) reoffended. The duration of follow-up reported by included studies ranged from 7 months to 22 years. Studies were from 13 countries: Canada (*n* = 7), the USA (*n* = 8), the UK (*n* = 5), Australia (*n* = 3), Sweden (*n* = 5), two each from Brazil, Germany and Italy and one each from Uganda, Korea, France, Japan and Spain. All but two investigations ascertained recidivism from register-based sources. The remaining studies used self-report measures.^51,60^

**Fig. 5:**
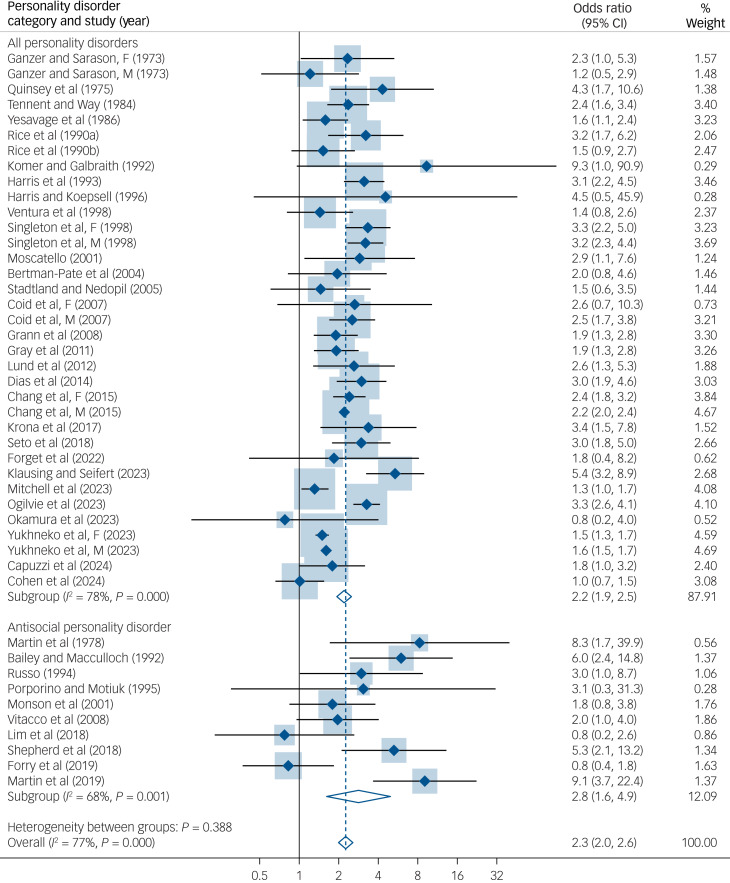
Risk estimates for recidivism in personality disorder-diagnosed individuals with a history of criminal behaviour compared with individuals with a history of criminal behaviour with or without other psychiatric disorders.^8^^,^^43–80^

Random-effects meta-analysis indicated the overall odds ratio for repeat offending associated with any personality disorder to be 2.3 (95% CI 2.0–2.6), with considerable heterogeneity between studies (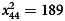, *I*^2^ = 77%, *P* ≤ 0.001) (Fig. 5). The odds ratio was similar when low-quality studies were excluded (odds ratio 2.3, 95% CI 2.0–2.6).

#### Antisocial personality disorder

There was a significant association between ASPD and recidivism (odds ratio 2.8, 95% CI 1.6–4.9), with moderate heterogeneity (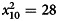, *I*^2^ = 68%, *P* = 0.001) (Fig. 5). The odds ratio for recidivism associated with ASPD was higher when low-quality studies were excluded (odds ratio 3.2, 95% CI 2.1–5.1). There was no significant difference in odds ratios in studies including individuals with ASPD compared with studies including individuals with any or other personality disorder. The population attributable risk of recidivism associated with ASPD was 54 per 1000 individuals, with 18.0% of reoffending incidents attributable to ASPD.

#### Violent versus general recidivism

There was no significant difference in risk estimates by the type of recidivism in all personality disorders (general recidivism: odds ratio 2.3, 95% CI 1.9–2.6; violent recidivism: odds ratio 2.5, 95% CI 2.0–3.2) and ASPD studies (general recidivism: odds ratio 2.8, 95% CI 1.6–4.9; violent recidivism: odds ratio 3.1, 95% CI 1.3–7.6).

#### Risk estimate by comparison groups

Most included studies (*n* = 33) compared recidivism risk in individuals with personality disorders with a history of criminal behaviour with recidivism risk in those with a history of criminal behaviour who had other psychiatric disorders. Four studies included individuals with a history of criminal behaviour without psychiatric disorders as the comparison group.^8,53,66,68^ One study included individuals without personality disorders (i.e. individuals with and without psychiatric disorders) in the comparison group.^63^ Five studies reported separate effect sizes respective to control groups with and without other psychiatric disorders.^46,49,60,75,80^ We found no significant difference in risk estimates from all personality disorder studies including individuals with other psychiatric disorders as a comparison group (odds ratio 2.3, 95% CI 2.1–2.7) versus individuals without other psychiatric disorders as comparison group (odds ratio 3.0, 95% CI 2.3–3.9). There was also no significant difference in recidivism risk estimates by comparison groups in ASPD samples: the odds ratio was 2.4 (95% CI 1.3–4.2) for studies using no psychiatric disorders as comparison group versus an odds ratio of 3.1 (95% CI 0.6–15.7) when other psychiatric disorders was the comparison group.

#### Other characteristics

In all personality disorders, cohort studies (odds ratio 2.4, 95% CI 2.1–2.7) (*k =* 35) and studies that ascertained recidivism outcomes using registers reported significantly higher risk estimates (odds ratio 2.3, 95% CI 2.0–2.6) (*k =* 43) than case–control studies (odds ratio 1.3, 95% CI 0.9–1.8) (*k =* 4) and studies using self-report measures (odds ratio 0.8, 95% CI 0.4–1.6) (*k* = 2). A similar pattern was found in ASPD samples (odds ratio, 3.8 [2.4, 6.2] *v.* 0.8 [0.4–1.6]). There was no significant difference in risk estimates by other study characteristics.

#### Meta-regression and publication bias

For all personality disorders, there were higher odds ratios in studies conducted in countries other than the USA and Scandinavian countries (β = 1.24, SE[β] = 0.12; *P* = 0.024) when variables were entered individually. However, these effects were no longer significant in multivariable regression. In ASPD, a similar pattern was found with higher odds ratios in registry data studies (univariable meta-regression: β = 0.21, SE[β] = 0.11; *P* = 0.018; multivariable meta-regression: β = 0.09, SE[β] = 0.05; *P* = 0.009). Egger's test found evidence of publication bias in studies on all personality disorders (including ASPD) (*t* = 2.40, *P* = 0.02), but not among ASPD studies (*t* = 0.63, *P* = 0.54).

## Discussion

We assessed the link between personality disorders and the risk of violence, antisocial behaviour and recidivism in updated systematic reviews and meta-analyses. The first review included data from 83 418 individuals diagnosed with personality disorders from 21 studies in 10 countries, nearly doubling the number of primary studies compared with a previous systematic review.^5^ Unlike the 2012 review, this update allowed us to stratify by individual personality disorder and provide more precision on risk estimates. We found a four- to five-fold increase in the odds of violence among individuals diagnosed with any personality disorder compared to general population controls. In ASPD, we found a seven-fold increase in the odds of violence. Overall, the violence risk associated with any personality disorder is similar to that associated with severe mental illness, while individuals with ASPD showed similar violence risk to individuals with substance misuse.^81^ In the second systematic review, we explored the risk of recidivism in 14 131 individuals diagnosed with personality disorders compared with individuals without personality disorders. In 39 studies, we found that individuals with personality disorders had a two to three times increased odds of reoffending compared with those without personality disorders. We also found that recidivism risk in ASPD was similar to any personality disorder, although the risk magnitude for any personality disorder may be partly driven by ASPD.

BPD was found to double the odds of violence compared to the general population. While some studies attributed the risk of violence and aggression associated with BPD to comorbidity with ASPD,^6,82^ BPD appears to be associated with an increased risk of violence independently, as most included studies controlled for ASPD.^26,27^ This is consistent with other work demonstrating a link between BPD and violence.^83,84^ One mechanism that explains this link is emotional dysregulation.^85^ The distinct risk estimates associated with BPD and ASPD may be explained by their specific internalising traits. Violence perpetrators with BPD were found to be more involved in reactive aggression, whereas individuals with ASPD engage in more instrumental and goal-directed aggression.^86^ However, how and to what extent these traits moderate the extent of violence risk is unclear. Given the conventional categorical classifications used for the diagnosis of ASPD and BPD, disentangling the overlapping traits (i.e. instability, impulsivity and emotional dysregulation) underlying their differential risk profiles remains challenging.^87^ Furthermore, DSM and ICD include violent behaviour (e.g. repeated physical fights) and unlawful acts (e.g. behaviours that are grounds for arrest or conflict with society) as indicators of ASPD, which likely contributed to the higher odds ratios found in ASPD studies.^88–90^ Thus, risk estimates for the link between all personality disorders and violence may be increased because of the contribution of studies where they sampled ASPD (and BPD, where impulsive behaviours are part of diagnostic criteria). We found some evidence for this – personality disorder samples that included those with ASPD had higher risk estimates. However, the analysis investigating personality disorder samples without ASPD showed increased violence risk. Consistent with this, studies with a low proportion of individuals with ASPD also showed increased risk. These findings suggest that the association between personality disorders and violence cannot be explained solely by the presence of ASPD in personality disorder samples. The lack of research on other individual personality disorders and violence, as well as the lack of longitudinal studies in the field, should be considered in interpreting the findings. We also found paranoid personality disorder to be associated with a one- to two-fold risk of violence, although data was available from just one study.^6^

Most included studies were of moderate and high quality, and sensitivity analyses found that excluding low-quality studies had only a small impact on the results. The majority of cohort studies on recidivism did not explicitly demonstrate whether previous reoffending was accounted for or absent at the start of follow-up, resulting in lower-quality scores.

Substance misuse is reported to be the strongest risk factor for violence across major psychiatric diagnostic categories.^81^ However, in the current review focusing on violence outcomes, only five out of the 16 included studies adjusted for substance misuse. Our analyses also found that SUD comorbidity increased the risk of violence in ASPD. Further research should compare violence and recidivism risks associated with ASPD and SUD to clarify their relative contributions, and further work should account for substance misuse. Moreover, in the three studies that examined BPD, the comparison group may have included personality disorders other than ASPD and BPD, which may have contributed to non-significant associations.^26,27,36^

### Limitations

Several limitations should be noted. First, the overall risk estimates should be interpreted with caution given the significant heterogeneity, particularly for violent outcomes. Second, most included studies on antisocial behaviour and violence relied on self-report measures from cross-sectional surveys with small and selected clinical samples. Compared to studies relying on registry data, the lower risk estimates for recidivism in ASPD reported in studies using self-report measures may reflect potential social desirability bias. The role of deceit, as one of the core symptoms of ASPD, may be relevant in the underreporting of criminality outcomes in the personality disorder group relative to controls.^89,91,92^ Third, publication bias in recidivism studies may be attributed to the small samples in some studies. Fourth, most included studies on violent outcomes used case–control or cross-sectional designs, which assessed personality disorder diagnosis and violent behaviour in participants simultaneously or retrospectively, and so the temporal order is uncertain. Furthermore, perpetration of violence is one criterion for ASPD diagnosis, which will complicate the findings as reverse causality is a possibility. We were unable to examine non-violent outcomes in ASPD to test for consistency in increased associations. Of the six cohort studies on violence,^30,31,33,36,37,41^ only three had a prospective design.^31,36,37^ The consistency of findings across different designs suggests that there are clear associations between personality disorders and violent outcomes, although causal inference will need triangulation of evidence with other designs (including treatment randomised controlled trials [RCTs]). Fifth, epidemiologic studies have reported narcissistic and obsessive–compulsive personality disorders to be the most prevalent personality disorders in the community setting,^93^ but we found a small amount of research on associations with violence. Finally, included studies were predominantly conducted in high-income countries.

### Implications

This review suggests that preventing violent and antisocial outcomes in people with personality disorders, particularly those with clinically significant borderline and antisocial traits, should be considered as part of routine clinical care. The risks are increased for all personality disorders, for the outcomes investigated (violence, antisocial behaviour and repeat offending) and the magnitude of the risk increases were not small. Prevention will be improved with better prediction and more evidence-based treatments, and potentially by managing substance misuse comorbidity. Predicting higher risk persons will allow for targeting of limited clinical resources, and provide for personalised management. Considering the wider move in the field towards focusing on traits and dimensions, treatments for violence and offending in personality disorders could include managing disinhibition (such as to being provoked or its perception), violent behaviour that is based on inflated sense of entitlement and consequences of emotion dysregulation (such as not thinking through the consequences of one's actions).^87,94^ Recommended treatments are currently psychological, although these do not have clear effectiveness for ASPD. England's National Institute for Health and Care Excellence (NICE) recommends group-based cognitive–behavioural therapy (CBT) and dialectical behaviour therapy for the management of antisocial behaviour and offending in ASPD and BPD.^95,96^ However, evidence on the efficacy in reducing aggressive behaviour and reconviction among individuals with personality disorders is inconclusive.^97–99^ Moreover, whether CBT-based interventions reduce reoffending in prisoners is unclear.^100^ As with personality disorders, dysfunctional inhibition and affective control mediate violent and aggressive behaviour in SUD.^101^ The success of psychosocial treatments addressing these traits (e.g. with a community-oriented group intervention) in reducing aggression and crime associated with SUD suggests their potential to reduce violence in personality disorders with comorbid substance misuse.^102^ There may also be a role of medication in preventing adverse outcomes: a large population-based study using within-individual designs to better account for confounding has shown a large association between antipsychotic prescription and lower rates of violent crime, which will need triangulation with trials.^103^ The role of beta-blockers and medications used for SUD needs further exploration in trials.^104,105^

In summary, links between personality disorders and increased risks of antisocial behaviour were consistent across outcomes, time periods and settings. Risks varied by individual personality disorder, with the highest observed in those with ASPD and with comorbid substance misuse. Improving identification and treatment for substance misuse could potentially reduce antisocial and violent outcomes in individuals with personality disorders.

## Supporting information

Chow et al. supplementary material Chow et al. supplementary material

## Data Availability

Data availability is not applicable to this article as no new data were created or analysed in this study.

## Analytic code availability

The analytic codes that support the meta-analyses in this review are available upon reasonable request.

## Research material availability

Tabular data in this review are available upon reasonable request.

## References

[1] Winsper C, Bilgin A, Thompson A, Marwaha S, Chanen AM, Singh SP, et al. The prevalence of personality disorders in the community: a global systematic review and meta-analysis. Br J Psychiatry 2020; 216(2): 69–78. 31298170 10.1192/bjp.2019.166

[2] Dixon-Gordon KL, Whalen DJ, Layden BK, Chapman AL. A systematic review of personality disorders and health outcomes. Can Psychol 2015; 56(2): 168–90. 26456998 10.1037/cap0000024 PMC4597592

[3] Fok ML, Hayes RD, Chang CK, Stewart R, Callard FJ, Moran P. Life expectancy at birth and all-cause mortality among people with personality disorder. J Psychosom Res 2012; 73(2): 104–7. 22789412 10.1016/j.jpsychores.2012.05.001

[4] Moran P, Romaniuk H, Coffey C, Chanen A, Degenhardt L, Borschmann R, et al. The influence of personality disorder on the future mental health and social adjustment of young adults: a population-based, longitudinal cohort study. Lancet Psychiatry 2016; 3(7): 636–45. 27342692 10.1016/S2215-0366(16)30029-3

[5] Yu R, Geddes JR, Fazel S. Personality disorders, violence, and antisocial behavior: a systematic review and meta-regression analysis. J Pers Disord 2012; 26(5): 775–92. 23013345 10.1521/pedi.2012.26.5.775

[6] Coid JW, Gonzalez R, Igoumenou A, Zhang T, Yang M, Bebbington P. Personality disorder and violence in the national household population of Britain. J Forensic Psychiatry Psychol 2017; 28(5): 620–38.

[7] Howard R, Hasin D, Stohl M. Substance use disorders and criminal justice contact among those with co-occurring antisocial and borderline personality disorders: findings from a nationally representative sample. Personal Ment Health 2021; 15(1): 40–8. 32588546 10.1002/pmh.1491

[8] Martin S, Zabala C, Del-Monte J, Graziani P, Aizpurua E, Barry TJ, et al. Examining the relationships between impulsivity, aggression, and recidivism for prisoners with antisocial personality disorder. Aggress Violent Behav 2019; 49: 101314.

[9] Wojciechowski T. The dual mediating roles of impulsivity and emotion regulation of the borderline personality disorder-violence relationship: a structural equation modeling approach. J Forensic Sci 2021; 66(6): 2329–39. 34286852 10.1111/1556-4029.14807

[10] Black DW, Gunter T, Allen J, Blum N, Arndt S, Wenman G, et al. Borderline personality disorder in male and female offenders newly committed to prison. Compr Psychiatry 2007; 48(5): 400–5. 17707246 10.1016/j.comppsych.2007.04.006

[11] Trestman RL, Ford J, Zhang W, Wiesbrock V. Current and lifetime psychiatric illness among inmates not identified as acutely mentally ill at intake in Connecticut's jails. J Am Acad Psychiatry Law 2007; 35(4): 490–500. 18086741

[12] Wetterborg D, Långström N, Andersson G, Enebrink P. Borderline personality disorder: prevalence and psychiatric comorbidity among male offenders on probation in Sweden. Compr Psychiatry 2015; 62: 63–70. 26343468 10.1016/j.comppsych.2015.06.014

[13] Moher D, Liberati A, Tetzlaff J, Altman DG. Preferred reporting items for systematic reviews and meta-analyses: the prisma statement. PLoS Med 2009; 6(7): e1000097. 19621072 10.1371/journal.pmed.1000097 PMC2707599

[14] Buchanan A, Moore KE, Pittman B, McKee SA. Psychosocial function, legal involvement and violence in mental disorder. Eur Psychiatry 2021; 64(1): e75. 34859762 10.1192/j.eurpsy.2021.2250 PMC8715282

[15] Nakic M, Stefanovics EA, Rhee TG, Rosenheck RA. Lifetime risk and correlates of incarceration in a nationally representative sample of U.S. Adults with non-substance-related mental illness. Soc Psychiatry Psychiatr Epidemiol 2022; 57(9): 1839–47. 34453553 10.1007/s00127-021-02158-x

[16] Borenstein M, Hedges LV, Higgins JP, Rothstein HR. A basic introduction to fixed-effect and random-effects models for meta-analysis. Res Synth Methods 2010; 1(2): 97–111. 26061376 10.1002/jrsm.12

[17] Higgins JPT, Green S. Cochrane Handbook for Systematic Reviews of Interventions Version 5.1.0 [Updated March 2011]. The Cochrane Collaboration, 2011.

[18] Egger M, Davey Smith G, Schneider M, Minder C. Bias in meta-analysis detected by a simple, graphical test. Br Med J 1997; 315(7109): 629–34. 9310563 10.1136/bmj.315.7109.629 PMC2127453

[19] Wells GA, Shea B, O'Connell D, Peterson J, Welch V, Losos M, Tugwell P. The Newcastle-Ottawa Scale (NOS) for Assessing the Quality of Nonrandomised Studies in Meta-Analyses. Ottawa Hospital Research Institute, 2021 (https://www.ohri.ca/programs/clinical_epidemiology/oxford.asp).

[20] Shrout PE, Fleiss JL. Intraclass correlations: uses in assessing rater reliability. Psychol Bull 1979; 86(2): 420–8. 18839484 10.1037//0033-2909.86.2.420

[21] Fleiss J. The Design and Analysis of Clinical Experiments. Wiley, 2011.

[22] Favril L, Yu R, Hawton K, Fazel S. Risk factors for self-harm in prison: a systematic review and meta-analysis. Lancet Psychiatry 2020; 7(8): 682–91. 32711709 10.1016/S2215-0366(20)30190-5 PMC7606912

[23] Cao RC, Chen XC, Yin L, Huang HL, Wan WZ, Li Y, et al. An epidemiologic survey and violent behavior analysis of antisocial personality disorder in young men in Chengdu. Fa Yi Xue Za Zhi 2022; 38(2): 239–45. 35899513 10.12116/j.issn.1004-5619.2020.100802

[24] Coid J, Yang M, Roberts A, Ullrich S, Moran P, Bebbington P, et al. Violence and psychiatric morbidity in a national household population – a report from the British household survey. Am J Epidemiol 2006; 164(12): 1199–208. 17032695 10.1093/aje/kwj339

[25] Elonheimo H, Niemelä S, Parkkola K, Multimäki P, Helenius H, Nuutila AM, et al. Police-registered offenses and psychiatric disorders among young males: the Finnish 'from a boy to a man' birth cohort study. Soc Psychiatry Psychiatr Epidemiol 2007; 42(6): 477–84. 17450452 10.1007/s00127-007-0192-1

[26] Gonzalez RA, Igoumenou A, Kallis C, Coid JW. Borderline personality disorder and violence in the UK population: categorical and dimensional trait assessment. BMC Psychiatry 2016; 16: 180. 27255770 10.1186/s12888-016-0885-7 PMC4891918

[27] Harford TC, Chen CM, Kerridge BT, Grant BF. Borderline personality disorder and violence toward self and others: a national study. J Pers Disord 2019; 33(5): 653–70. 30307827 10.1521/pedi_2018_32_361 PMC10225867

[28] Hodgins S, Mednick SA, Brennan PA, Schulsinger F, Engberg M. Mental disorder and crime. Evidence from a danish birth cohort. Arch Gen Psychiatry 1996; 53(6): 489–96. 8639031 10.1001/archpsyc.1996.01830060031004

[29] Johnson JG, Cohen P, Smailes E, Kasen S, Oldham JM, Skodol AE, et al. Adolescent personality disorders associated with violence and criminal behavior during adolescence and early adulthood. Am J Psychiatry 2000; 157(9): 1406–12. 10964855 10.1176/appi.ajp.157.9.1406

[30] Mok PLH, Walter F, Carr MJ, Antonsen S, Kapur N, Steeg S, et al. Absolute risks of self-harm and interpersonal violence by diagnostic category following first discharge from inpatient psychiatric care. Eur Psychiatry 2023; 66(1): e13. 36649931 10.1192/j.eurpsy.2022.2352 PMC9970150

[31] Monahan J, Appelbaum PS. Diagnostically based clues from the MacArthur violence risk assessment study. In Violence Among the Mentally III: Effective Treatments and Management Strategies (ed S Hodgins): 19–34. Kluwer Academic Publishers, 2000.

[32] Ortmann J. Psykisk afvigelse og kriminel adfærd: En undersøgelse af 11.533 mænd født i 1953 i det metropolitane område københavn. [Mental disorder and criminal behaviour. An investigation of 11,533 men born in 1953 in Copenhagen metropolitan area]. Justitsministeriet, 1981.

[33] Sariaslan A, Arseneault L, Larsson H, Lichtenstein P, Fazel S. Risk of subjection to violence and perpetration of violence in persons with psychiatric disorders in Sweden. JAMA Psychiatry 2020; 77(4): 359–67. 31940015 10.1001/jamapsychiatry.2019.4275 PMC6990843

[34] Stueve A, Link BG. Violence and psychiatric disorders: results from an epidemiological study of young adults in Israel. Psychiatr Q 1997; 68(4): 327–42. 9355133 10.1023/a:1025443014158

[35] Swanson MC, Bland RC, Newman SC. Epidemiology of psychiatric disorders in Edmonton. Antisocial personality disorders. Acta Psychiatr Scand Suppl 1994; 376: 63–70. 8178687

[36] Tate AE, Sahlin H, Liu S, Lu Y, Lundstrom S, Larsson H, et al. Borderline personality disorder: associations with psychiatric disorders, somatic illnesses, trauma, and adverse behaviors. Mol Psychiatry 2022; 27(5): 2514–21. 35304564 10.1038/s41380-022-01503-z PMC9135625

[37] ten Have M, de Graaf R, van Weeghel J, van Dorsselaer S. The association between common mental disorders and violence: to what extent is it influenced by prior victimization, negative life events and low levels of social support? Psychol Med 2014; 44(7): 1485–98. 24001369 10.1017/S0033291713002262

[38] Casiano H, Hensel JM, Chartier MJ, Ekuma O, MacWilliam L, Mota N, et al. The intersection between criminal accusations, victimization, and mental disorders: a Canadian population-based study. Can J Psychiatry 2020; 65(7): 492–501. 32363932 10.1177/0706743720919660 PMC7298584

[39] Durbin JR, Pasewark RA, Albers D. Criminality and mental illness: a study of arrest rates in a rural state. Am J Psychiatry 1977; 134(1): 80–3. 831548 10.1176/ajp.134.1.80

[40] Modestin J, Ammann R. Mental disorders and criminal behaviour. Br J Psychiatry 1995; 166(5): 667–75. 7620755 10.1192/bjp.166.5.667

[41] Steadman HJ, Cocozza JJ, Melick ME. Explaining the increased arrest rate among mental patients: the changing clientele of state hospitals. Am J Psychiatry 1978; 135(7): 816–20. 665793 10.1176/ajp.135.7.816

[42] Tsai J, Edwards E, Cao X, Finlay AK. Disentangling associations between military service, race, and incarceration in the U.S. Population. Psychol Serv 2022; 19(3): 431–42. 35878068 10.1037/ser0000537

[43] Bailey J, Macculloch M. Characteristics of 112 cases discharged directly to the community from a new special hospital and some comparisons of performance. J Forensic Psychiatry 1992; 3(1): 91–112.

[44] Bertman-Pate LJ, Burnett DM, Thompson JW, Calhoun CJ Jr., Deland S, Fryou RM. The New Orleans forensic aftercare clinic: a seven year review of hospital discharged and jail diverted clients. Behav Sci Law 2004; 22(1): 159–69. 14963885 10.1002/bsl.575

[45] Capuzzi E, Caldiroli A, Auxilia AM, Capellazzi M, Tagliabue I, Manzoni A, et al. Which sociodemographic and clinical characteristics are associated with recurrent incarcerations in adult male people who are incarcerated? A cross-sectional study. J Forensic Psychiatry Psychol 2024; 35(2): 147–70.

[46] Chang Z, Larsson H, Lichtenstein P, Fazel S. Psychiatric disorders and violent reoffending: a national cohort study of convicted prisoners in Sweden. Lancet Psychiatry 2015; 2(10): 891–900. 26342957 10.1016/S2215-0366(15)00234-5 PMC4629414

[47] Cohen TR, Fronk GE, Kiehl KA, Curtin JJ, Koenigs M. Clarifying the relationship between mental illness and recidivism using machine learning: a retrospective study. PLoS One 2024; 19: e0297448. 38394314 10.1371/journal.pone.0297448 PMC10890739

[48] Coid J, Hickey N, Kahtan N, Zhang T, Yang M. Patients discharged from medium secure forensic psychiatry services: reconvictions and risk factors. Br J Psychiatry 2007; 190: 223–9. 17329742 10.1192/bjp.bp.105.018788

[49] Dias A, Serafim A, Barros D. Prevalence of mental disorders and recidivism in young offenders. Psicol Reflexão e Crítica 2014; 27: 317–22.

[50] Forget K, Gagné P, Douyon SS, Poirier C, LeBlanc J, Bilodeau MC, et al. Psychiatric relapse and criminal recidivism of individuals found not criminally responsible on account of mental disorder after absolute discharge. Can J Psychiatry 2022; 67(11): 864–6. 35929073 10.1177/07067437221116983 PMC9561695

[51] Forry JB, Kirabira J, Ashaba S, Rukundo GZ. Crime, recidivism and mental disorders among prisoners in mbarara municipality, southwestern Uganda. Int J Law Psychiatry 2019; 62: 1–6. 30616843 10.1016/j.ijlp.2018.10.006

[52] Ganzer VJ, Sarason IG. Variables associated with recidivism among juvenile delinquents. J Consul Clin Psychol 1973; 40(1): 1–5. 10.1037/h0034012 4688674

[53] Grann M, Danesh J, Fazel S. The association between psychiatric diagnosis and violent re-offending in adult offenders in the community. BMC Psychiatry 2008; 8(1): 92. 19032787 10.1186/1471-244X-8-92 PMC2611986

[54] Gray NS, Taylor J, Snowden RJ. Predicting violence using structured professional judgment in patients with different mental and behavioral disorders. Psychiatry Res 2011; 187(1–2): 248–53. 21095013 10.1016/j.psychres.2010.10.011

[55] Harris GT, Rice ME, Quinsey VL. Violent recidivism of mentally disordered offenders: the development of a statistical prediction instrument. Crim Just Behav 1993; 20: 315–35.

[56] Harris V, Koepsell TD. Criminal recidivism in mentally ill offenders: a pilot study. Bull Am Acad Psychiatry Law 1996; 24(2): 177–86. 8807158

[57] Klausing H, Seifert D. Rückfallverläufe von entlassenen Maßregelvollzugspatienten (§ 63 StGB) differenziert nach Diagnosegruppen [Recidivism of discharged forensic patient (§ 63 stgb) differentiated according to diagnosis groups]. Psychiatr Prax 2023; 50(4): 189–95. 35790177 10.1055/a-1855-9773

[58] Komer B, Galbraith D. Recidivism among individuals detained under a warrant of the lieutenant-governor living in the community. Can J Psychiatry 1992; 37(10): 694–8. 1473074 10.1177/070674379203701004

[59] Krona H, Nyman M, Andreasson H, Vicencio N, Anckarsäter H, Wallinius M, et al. Mentally disordered offenders in Sweden: differentiating recidivists from non-recidivists in a 10-year follow-up study. Nord J Psychiatry 2017; 71(2): 102–9. 27701993 10.1080/08039488.2016.1236400

[60] Lim Y, Park EJ, Kim B. Psychiatric disorders and recidivism among Korean adolescents on probation or parole. Psychiatry Invest 2018; 15(6): 561–7. 10.30773/pi.2017.11.30.1 PMC6018139 29788699

[61] Lund C, Forsman A, Anckarsäter H, Nilsson T. Early criminal recidivism among mentally disordered offenders. Int J Offender Ther Comp Criminol 2012; 56(5): 749–68. 21803759 10.1177/0306624X11411677

[62] Martin RL, Cloninger CR, Guze SB. Female criminality and the prediction of recidivism: a prospective six-year follow-up. Arch Gen Psychiatry 1978; 35(2): 207–14. 623507 10.1001/archpsyc.1978.01770260085010

[63] Mitchell RJ, Burns N, Glozier N, Nielssen O. Homelessness and predictors of criminal reoffending: a retrospective cohort study. Crim Behav Ment Health 2023; 33(4): 261–75. 37269064 10.1002/cbm.2298

[64] Monson CM, Gunnin DD, Fogel MH, Kyle LL. Stopping (or slowing) the revolving door: factors related to ngri acquittees’ maintenance of a conditional release. Law Hum Behav 2001; 25(3): 257–67. 11480803 10.1023/a:1010745927735

[65] Moscatello R. Recidiva criminal em 100 internos do manicômio judiciário de franco da rocha. Braz J Psychiatry 2001; 23(1): 34–5.

[66] Ogilvie JM, Tzoumakis S, Thompson C, Allard T, Dennison S, Kisely S, et al. Psychiatric illness and the risk of reoffending: recurrent event analysis for an Australian birth cohort. BMC Psychiatry 2023; 23(1): 355. 37221485 10.1186/s12888-023-04839-0 PMC10207651

[67] Okamura M, Okada T, Okumura Y. Recidivism among prisoners with severe mental disorders. Heliyon 2023; 9(6): e17007. 37484360 10.1016/j.heliyon.2023.e17007 PMC10361118

[68] Porporino FJ, Motiuk LL. The prison careers of mentally disordered offenders. Int J Law Psychiatry 1995; 18(1): 29–44. 7759187 10.1016/0160-2527(94)00025-5

[69] Quinsey VL, Warneford A, Pruesse M, Link N. Released Oak Ridge patients: a follow-up study of review board discharges. Br J Criminol 1975; 15(3): 264–79.

[70] Rice ME, Harris GT, Lang C, Bell V. Recidivism among male insanity acquittees. J Psychiatry Law 1990; 18(3–4): 379–403.

[71] Russo G. Follow-up of 91 mentally ill criminals discharged from the maximum security hospital in Barcelona P.G. Int J Law Psychiatry 1994; 17(3): 279–301. 7995687 10.1016/0160-2527(94)90031-0

[72] Seto MC, Charette Y, Nicholls TL, Crocker AG. Individual, service, and neighborhood predictors of aggression among persons with mental disorders. Crim Just Behav 2018; 45(7): 929–48.

[73] Shepherd SM, Campbell RE, Ogloff JRP. Psychopathy, antisocial personality disorder, and reconviction in an Australian sample of forensic patients. Int J Offender Ther Comp Criminol 2018; 62(3): 609–28. 27288398 10.1177/0306624X16653193

[74] Singleton N, Meltzer H, Gatward R. Psychiatric morbidity among prisoners in England and Wales. Stationery Office, 1998.

[75] Stadtland C, Nedopil N. Psychiatrische erkrankungen und die prognose krimineller rückfälligkeit. [psychiatric disorders and the prognosis for criminal recidivism.]. Nervenarzt 2005; 76(11): 1402–11. 15448915 10.1007/s00115-004-1808-2

[76] Tennent G, Way C. The English special hospital – a 12–17 year follow-up study: a comparison of violent and non-violent re-offenders and non-offenders. Med Sci Law 1984; 24(2): 81–91. 6727615 10.1177/002580248402400204

[77] Ventura LA, Cassel CA, Jacoby JE, Huang B. Case management and recidivism of mentally ill persons released from jail. Psychiatr Serv 1998; 49(10): 1330–7. 9779904 10.1176/ps.49.10.1330

[78] Vitacco MJ, Van Rybroek GJ, Erickson SK, Rogstad JE, Tripp A, Harris L, et al. Developing services for insanity acquittees conditionally released into the community: maximizing success and minimizing recidivism. Psychol Serv 2008; 5(2): 118–25.

[79] Yesavage JA, Benezech M, Larrieu-Arguille R, Bourgeois M, Tanke E, Rager P, et al. Recidivism of the criminally insane in France: a 22-year follow-up. J Clin Psychiatry 1986; 47(9): 465–6. 3745128

[80] Yukhnenko D, Blackwood N, Lichtenstein P, Fazel S. Psychiatric disorders and reoffending risk in individuals with community sentences in Sweden: a national cohort study. Lancet Public Health 2023; 8(2): e119–e29. 36669512 10.1016/S2468-2667(22)00312-7 PMC10914666

[81] Fazel S, Smith EN, Chang Z, Geddes JR. Risk factors for interpersonal violence: an umbrella review of meta-analyses. Br J Psychiatry 2018; 213(4): 609–14. 30058516 10.1192/bjp.2018.145 PMC6157722

[82] Howard RC, Khalifa N, Duggan C. Antisocial personality disorder comorbid with borderline pathology and psychopathy is associated with severe violence in a forensic sample. J Forensic Psychiatry Psychol 2014; 25(6): 658–72.

[83] Newhill CE, Eack SM, Mulvey EP. Violent behavior in borderline personality. J Pers Disord 2009; 23(6): 541–54. 20001173 10.1521/pedi.2009.23.6.541

[84] Newhill CE, Eack SM, Mulvey EP. A growth curve analysis of emotion dysregulation as a mediator for violence in individuals with and without borderline personality disorder. J Pers Disord 2012; 26(3): 452–67. 22686232 10.1521/pedi.2012.26.3.452

[85] Scott LN, Stepp SD, Pilkonis PA. Prospective associations between features of borderline personality disorder, emotion dysregulation, and aggression. Pers Disord 2014; 5(3): 278–88. 10.1037/per0000070 PMC4099305 24635753

[86] Gilbert F, Daffern M. Illuminating the relationship between personality disorder and violence: contributions of the general aggression model. Psychol Violence 2011; 1(3): 230–44.

[87] Lowenstein J, Purvis C, Rose K. A systematic review on the relationship between antisocial, borderline and narcissistic personality disorder diagnostic traits and risk of violence to others in a clinical and forensic sample. Borderline Pers Disord Emot Dysregul 2016; 3: 14. 10.1186/s40479-016-0046-0 PMC5062934 27777779

[88] American Psychiatric Association. Diagnostic and Statistical Manual of Mental Disorders (3rd edn). American Psychiatric Publishing, Inc., 1988.

[89] American Psychiatric Association. Diagnostic and Statistical Manual of Mental Disorders (4th edn). American Psychiatric Publishing, Inc., 1994.

[90] World Health Organization (WHO). The ICD-10 Classification of Mental and Behavioural Disorders. WHO, 1993.

[91] American Psychiatric Association. Diagnostic and Statistical Manual of Mental Disorders (5th edn). American Psychiatric Publishing, Inc., 2013.

[92] Jiang W, Liu H, Liao J, Ma X, Rong P, Tang Y, et al. A functional mri study of deception among offenders with antisocial personality disorders. Neuroscience 2013; 244: 90–8. 23578713 10.1016/j.neuroscience.2013.03.055

[93] Sansone RA, Sansone LA. Personality disorders: a nation-based perspective on prevalence. Innov Clin Neurosci 2011; 8(4): 13–8. PMC3105841 21637629

[94] Fisher S, Hall G. ‘If you show a bit of violence they learn real quick’: measuring entitlement in violent offenders. Psychiatry Psychol Law 2011; 18(4): 588–98.

[95] National Institute for Health and Care Excellence (NICE). Antisocial Personality Disorder: Prevention and Management (NICE guideline no. 77). NICE, 2009 (https://www.nice.org.uk/guidance/cg77). 32208571

[96] National Institute for Health and Care Excellence (NICE). Borderline Personality Disorder: Recognition and Management (NICE guideline no. 78). NICE, 2009 (https://www.nice.org.uk/guidance/cg78). 39480982

[97] Ciesinski NK, Sorgi-Wilson KM, Cheung JC, Chen EY, McCloskey MS. The effect of dialectical behavior therapy on anger and aggressive behavior: a systematic review with meta-analysis. Behav Res Ther 2022; 154: 104122. 35609374 10.1016/j.brat.2022.104122

[98] Khalifa NR, Gibbon S, Vollm BA, Cheung NH, McCarthy L. Pharmacological interventions for antisocial personality disorder. Cochrane Database Syst Rev 2020; 9(9): CD007667. 32880105 10.1002/14651858.CD007667.pub3 PMC8094881

[99] Rampling J, Furtado V, Winsper C, Marwaha S, Lucca G, Livanou M, et al. Non-pharmacological interventions for reducing aggression and violence in serious mental illness: a systematic review and narrative synthesis. Eur Psychiatry 2016; 34: 17–28. 26928342 10.1016/j.eurpsy.2016.01.2422

[100] Beaudry G, Yu R, Perry AE, Fazel S. Effectiveness of psychological interventions in prison to reduce recidivism: a systematic review and meta-analysis of randomised controlled trials. Lancet Psychiatry 2021; 8(9): 759–73. 34419185 10.1016/S2215-0366(21)00170-X PMC8376657

[101] Tomlinson M, Brown M, Hoaken P. Recreational drug use and human aggressive behavior: a comprehensive review since 2003. Aggress Violent Behav 2016; 27: 9–29.

[102] Thekkumkara SN, Jagannathan A, Muliyala KP, Murthy P. Psychosocial interventions for prisoners with mental and substance use disorders: a systematic review. Indian J Psychol Med 2022; 44(3): 211–7. 35656427 10.1177/02537176211061655 PMC9125461

[103] Herttua K, Crawford M, Paljarvi T, Fazel S. Associations between antipsychotics and risk of violent crimes and suicidal behaviour in personality disorder. Evid Based Ment Health 2022; 25(e1): e58–64. 36283800 10.1136/ebmental-2022-300493 PMC9811101

[104] Molero Y, Zetterqvist J, Binswanger IA, Hellner C, Larsson H, Fazel S. Medications for alcohol and opioid use disorders and risk of suicidal behavior, accidental overdoses, and crime. Am J Psychiatry 2018; 175(10): 970–8. 30068260 10.1176/appi.ajp.2018.17101112 PMC6169735

[105] Molero Y, Kaddoura S, Kuja-Halkola R, Larsson H, Lichtenstein P, D'Onofrio BM, et al. Associations between β-blockers and psychiatric and behavioural outcomes: a population-based cohort study of 1.4 million individuals in Sweden. PLoS Med 2023; 20(1): e1004164. 36719888 10.1371/journal.pmed.1004164 PMC9888684

