# Supplementary material for: Personality disorders, violence and antisocial behaviour: updated systematic review and meta-regression analysis
Source: Br J Psychiatry. 2025 Jul;227(1):481–91. doi: 10.1192/bjp.2024.226 (PMC12278050; doi:10.1192/bjp.2024.226)
Supplement: Chow et al. supplementary material [file S0007125024002265sup001.docx]

Personality disorders, violence, and antisocial behaviour: An updated systematic review and meta-regression analysis

Rachel T. S. Chow, Rongqin Yu, John R. Geddes, and Seena Fazel

Supplementary materials

**APPENDICES**

**Appendix A. PRISMA checklist**

| **Section and Topic** | **Item #** | **Checklist item** | **Location where item is reported** |
| --- | --- | --- | --- |
| **TITLE** | | |  |
| Title | 1 | Identify the report as a systematic review. | Page 1 |
| **ABSTRACT** | | |  |
| Abstract | 2 | See the PRISMA 2020 for Abstracts checklist. | Page 2 |
| **INTRODUCTION** | | |  |
| Rationale | 3 | Describe the rationale for the review in the context of existing knowledge. | Page 3 |
| Objectives | 4 | Provide an explicit statement of the objective(s) or question(s) the review addresses. | Page 3 |
| **METHODS** | | |  |
| Eligibility criteria | 5 | Specify the inclusion and exclusion criteria for the review and how studies were grouped for the syntheses. | Page 5 |
| Information sources | 6 | Specify all databases, registers, websites, organisations, reference lists and other sources searched or consulted to identify studies. Specify the date when each source was last searched or consulted. | Page 4 |
| Search strategy | 7 | Present the full search strategies for all databases, registers and websites, including any filters and limits used. | Page 4 |
| Selection process | 8 | Specify the methods used to decide whether a study met the inclusion criteria of the review, including how many reviewers screened each record and each report retrieved, whether they worked independently, and if applicable, details of automation tools used in the process. | Page 5 |
| Data collection process | 9 | Specify the methods used to collect data from reports, including how many reviewers collected data from each report, whether they worked independently, any processes for obtaining or confirming data from study investigators, and if applicable, details of automation tools used in the process. | Page 5-6 |
| Data items | 10a | List and define all outcomes for which data were sought. Specify whether all results that were compatible with each outcome domain in each study were sought (e.g. for all measures, time points, analyses), and if not, the methods used to decide which results to collect. | Page 5-6 |
|  | 10b | List and define all other variables for which data were sought (e.g. participant and intervention characteristics, funding sources). Describe any assumptions made about any missing or unclear information. | Page 5-6 |
| Study risk of bias assessment | 11 | Specify the methods used to assess risk of bias in the included studies, including details of the tool(s) used, how many reviewers assessed each study and whether they worked independently, and if applicable, details of automation tools used in the process. | Page 7-8 |
| Effect measures | 12 | Specify for each outcome the effect measure(s) (e.g. risk ratio, mean difference) used in the synthesis or presentation of results. | Page 6 |
| Synthesis methods | 13a | Describe the processes used to decide which studies were eligible for each synthesis (e.g. tabulating the study intervention characteristics and comparing against the planned groups for each synthesis (item #5)). | Page 5-6 |
|  | 13b | Describe any methods required to prepare the data for presentation or synthesis, such as handling of missing summary statistics, or data conversions. | Page 6 |
|  | 13c | Describe any methods used to tabulate or visually display results of individual studies and syntheses. | Page 6 |
|  | 13d | Describe any methods used to synthesize results and provide a rationale for the choice(s). If meta-analysis was performed, describe the model(s), method(s) to identify the presence and extent of statistical heterogeneity, and software package(s) used. | Page 6-7 |
|  | 13e | Describe any methods used to explore possible causes of heterogeneity among study results (e.g. subgroup analysis, meta-regression). | Page 6-7 |
|  | 13f | Describe any sensitivity analyses conducted to assess robustness of the synthesized results. | Page 7 |
| Reporting bias assessment | 14 | Describe any methods used to assess risk of bias due to missing results in a synthesis (arising from reporting biases). | N/A |
| Certainty assessment | 15 | Describe any methods used to assess certainty (or confidence) in the body of evidence for an outcome. | Page 7-8 |
| **RESULTS** | | |  |
| Study selection | 16a | Describe the results of the search and selection process, from the number of records identified in the search to the number of studies included in the review, ideally using a flow diagram. | Page 8, Figure 1 |
|  | 16b | Cite studies that might appear to meet the inclusion criteria, but which were excluded, and explain why they were excluded. | Page 5 |
| Study characteristics | 17 | Cite each included study and present its characteristics. | Page 8, 12, Appendix B |
| Risk of bias in studies | 18 | Present assessments of risk of bias for each included study. | Page 8 |
| Results of individual studies | 19 | For all outcomes, present, for each study: (a) summary statistics for each group (where appropriate) and (b) an effect estimate and its precision (e.g. confidence/credible interval), ideally using structured tables or plots. | Figures 2-5, Appendix Figures B.1-2 |
| Results of syntheses | 20a | For each synthesis, briefly summarise the characteristics and risk of bias among contributing studies. | Page 9-10, 12-13 |
|  | 20b | Present results of all statistical syntheses conducted. If meta-analysis was done, present for each the summary estimate and its precision (e.g. confidence/credible interval) and measures of statistical heterogeneity. If comparing groups, describe the direction of the effect. | Page 9-14 |
|  | 20c | Present results of all investigations of possible causes of heterogeneity among study results. | Page 10-12, 13-14 |
|  | 20d | Present results of all sensitivity analyses conducted to assess the robustness of the synthesized results. | Page 9, 13 |
| Reporting biases | 21 | Present assessments of risk of bias due to missing results (arising from reporting biases) for each synthesis assessed. | N/A |
| Certainty of evidence | 22 | Present assessments of certainty (or confidence) in the body of evidence for each outcome assessed. | Page 8 |
| **DISCUSSION** | | |  |
| Discussion | 23a | Provide a general interpretation of the results in the context of other evidence. | Page 15-17 |
|  | 23b | Discuss any limitations of the evidence included in the review. | Page 17-18 |
|  | 23c | Discuss any limitations of the review processes used. | Page 17-18 |
|  | 23d | Discuss implications of the results for practice, policy, and future research. | Page 18-19 |
| **OTHER INFORMATION** | | |  |
| Registration and protocol | 24a | Provide registration information for the review, including register name and registration number, or state that the review was not registered. | Page 4 |
|  | 24b | Indicate where the review protocol can be accessed, or state that a protocol was not prepared. | Page 4 |
|  | 24c | Describe and explain any amendments to information provided at registration or in the protocol. | N/A |
| Support | 25 | Describe sources of financial or non-financial support for the review, and the role of the funders or sponsors in the review. | Page 20 |
| Competing interests | 26 | Declare any competing interests of review authors. | Page 20 |
| Availability of data, code and other materials | 27 | Report which of the following are publicly available and where they can be found: template data collection forms; data extracted from included studies; data used for all analyses; analytic code; any other materials used in the review. | Page 21 |

**Appendix B. Characteristics of Included Studies**

**Table B.1. Characteristics of Included Studies on the Risk of Antisocial Behaviour and Violence in PDs**

| Study | Country | Study type | PDs (*N*) | Controls (*N*) | Cases | Comparison Group | Sex |
| --- | --- | --- | --- | --- | --- | --- | --- |
| Additional studies identified in this updated review | | | | | | | |
| ten Have et al. (2014) | Netherlands | Cohort | 128 | * | ASPD | GP without psychiatric disorders | Mixed |
| Gonzalez et al. (2016) | UK | Cross-sectional | 219 | 14534 | BPD | GP without BPD | Mixed |
| Coid et al. (2017) | UK | Cross-sectional | 73 | * | ASPD | GP | Female |
|  |  |  | 268 |  | ASPD |  | Male |
|  |  |  | 386^a^ |  | AvPD |  | Mixed |
|  |  |  | 103^a^ |  | DPD |  | Mixed |
|  |  |  | 59^a^ |  | HPD |  | Mixed |
|  |  |  | 40^a^ |  | NPD |  | Mixed |
|  |  |  | 856^a^ |  | OCPD |  | Mixed |
|  |  |  | 602^a^ |  | PPD |  | Mixed |
|  |  |  | 1081^a^ |  | ScPD |  | Mixed |
|  |  |  | 224^a^ |  | StPD |  | Mixed |
| Harford et al. (2019) | USA | Cross-sectional | 4301 | 32008 | BPD | GP without BPD | Mixed |
| Casiano et al. (2020) | Canada | Cross-sectional | 6342 | 486289 | All PDs | GP without psychiatric disorders | Mixed |
| Sariaslan et al. (2020) | Sweden | Cohort | 29713 | 2504190 | All PDs | GP without psychiatric disorders | Mixed |
| Cao et al. (2022) | China | Cross-sectional | 239 | 3805 | ASPD | GP without ASPD | Male |
| Tate et al. (2022) | Sweden | Cohort | 12175 | 1957664 | BPD | GP without BPD | Mixed |
| Tsai et al. (2022) | USA | Cross-sectional | 932 | 29405 | ASPD | GP | Mixed |
|  |  |  | 4287 |  | BPD | GP | Mixed |
|  |  |  | 2429 |  | StPD | GP | Mixed |
| Mok et al., F (2023) | Denmark | Cohort | 5686 | 802925 | All PDs | GP | Female |
| Mok et al., M (2023) | Denmark | Cohort | 3268 | 770125 | All PDs | GP | Male |
| Studies identified in the 2012 review | | | | | | | |
| Durbin et al. (1977) | USA | Cross-sectional | 29 | 93818 | All PDs | GP | Male |
| Steadman et al. (1978) | USA | Cohort | 56 | 12320540 | All PDs | GP | Male |
| Ortmann (1981) | Denmark | Case-control | 135 | 10974 | All PDs | GP | Male |
| Swanson (1994) | Canada | Cross-sectional | 104 | 3154 | ASPD | GP | Mixed |
| Modestin & Ammannn, F (1995) | Switzerland | Cross-sectional | 141 | 578 | All PDs | GP | Female |
| Modestin & Ammannn, M (1995) | Switzerland | Cross-sectional | 203 | 687 | All PDs | GP | Male |
| Hodgins et al., F (1996) | Denmark | Cross-sectional | 3553 | 147367 | ASPD without comorbidity of major mental health disorders | GP without psychiatric disorders | Female |
| Hodgins et al., M (1996) | Denmark | Cross-sectional | 3069 | 155580 | ASPD without comorbidity of major mental health disorders | GP without psychiatric disorders | Male |
| Stueve & Link (1997) | Israel | Cross-sectional | 49 | 1688 | ASPD only | GP without psychiatric disorders | Mixed |
| Monahan & Appelbaum (2000) | USA | Cohort | 20 | 519 | All PDs without comorbidity of other psychiatric disorders | GP | Male |
| Johnson et al. (2000) | USA | Case-control | 103 | 614 | All PDs (excluding ASPD) | GP | Mixed |
| Coid et al., F (2006) | UK | Cross-sectional | 1135 | 2603 | All PDs | GP without psychiatric disorders | Female |
| Coid et al., M (2006) | UK | Cross-sectional | 1337 | 2365 | All PDs | GP without psychiatric disorders | Male |
| Elonheimo et al. (2007) | Finland | Case-control | 73 | 2429 | ASPD | GP without psychiatric disorders | Male |

(Table 1 continues)

| Mean age | Diagnostic Criteria | Ascertainment of Outcomes | Adjustment^b^ | Definition of Antisocial behaviour |
| --- | --- | --- | --- | --- |
| 18-64 | DSM-IV | Self-report | Yes: adjusted for sex, age, education, ethnicity, living situation, employment situation, household income, the number of days between participants’ interviews at both waves of data collection, victimisation at childhood, number of negative life events, any social support, and the victimisation in adulthood experienced by any person in general | Violent behaviour towards any person(s) in general: kicking, biting, hitting, and intention to wound with an object or hot water |
| ≥16 | DSM-IV | Self-report | Yes: adjusted for sex, age, marital status, social class, ethnicity, ASPD, drug and alcohol dependence, psychosis, and anxiety disorder | Violent behaviour: physical fight and assault, weapon use, and hitting |
| 16-74 | DSM-IV | Self-report | Yes: adjusted for age, marital status, employment status, social class and ethnicity, comorbid psychopathology: anxiety (including generalised anxiety disorder, mixed anxiety and depression, phobias, obsessive compulsive disorder, and panic disorder), depressive episode, substance dependence, positive psychosis screening, and PDs other than the one primarily investigated in a particular association  *Information on this row applies to all reported associations in Coid et al. (2017)* | Violent behaviour: physical fight and assault, weapon use, and hitting |
| ≥18 | DSM-5 | Self-report | Yes: adjusted for sex, age, race, education, marital status, family income, other PDs (ASPD and StPD), alcohol use disorder, tobacco use disorder, cannabis use disorder, opioid use disorder, other drug use disorders, mood disorder, panic disorder, agoraphobia, specific phobia, social phobia, generalised anxiety disorder, post-traumatic stress disorder | Violent behaviour: physical fight, hitting, physically hurting someone, exchanging blows in fights and weapon use |
| 18-64 | ICD-10-CA  ICD-9-CM | Register | No | All crimes: administrative offences, drug-related offences, offences against person or property, assaults, and sexual and violent offences |
| 21.8 | ICD-8  ICD-9  ICD-10 | Register | No | Violent behaviour: violent crime convictions |
| 18-34 | DSM-IV | Self-report | Yes: adjusted for age, education, ethnicity, employment, marital status, geographical location, psychiatric disorders, and substance use | Violent behaviour: physical fight, hitting and physically hurting someone |
| 29.7 | ICD-9  ICD-10 | Register | No | All crimes: criminal convictions of violent (assault, threats of violence, and committing bodily injury) and non-violent (petty theft, fake passports or identifications, and property damage) nature |
| 45.5 | DSM-5 | Self-report | Yes: sociodemographic information (e.g. male gender, race, veteran status, education), physical and mental health, adverse life experiences, and perceived social support  *Information on this row applies to all reported associations in Tsai et al. (2023)* | All crimes: lifetime incarceration |
| * | ICD-10 | Register | No | Violent behaviour: convictions for physical violence (e.g., homicide, assault, robbery, aggravated burglary or arson, possessing weapons, kidnapping), threats of violence, coercision (e.g. human trafficking), terrorism, and sexual offences |
| * | ICD-10 | Register | No | Violent behaviour: convictions for physical violence (e.g., homicide, assault, robbery, aggravated burglary or arson, possessing weapons, kidnapping), threats of violence, coercision (e.g. human trafficking), terrorism, and sexual offences |
| Studies identified in the 2012 review | | | | |
| 18-64 | * | Register | No | All crimes: public intoxication, driving while intoxicated, disorderly conduct, traffic violations, and suspicion |
| 35 | * | Register | No | All crimes: any arrest |
| 25 | ICD-8 | Register | No | Violent behaviour: violent offences excluding sexual offences |
| * | DSM-III | Self-report | Yes: adjusted for household size, age, and sex distribution | Violent behaviour: any physical violence |
| 43.8 | ICD-9 | Register | Yes: matched sex, age, marital status, social class, and size of the community | All crimes |
| 39.4 | ICD-9 | Register | Yes: matched sex, age, marital status, social class, and size of the community | All crimes |
| > 35 | ICD-8 | Register | No | All crimes: violent offenses, theft of all kinds, fraud, vandalism, traffic offenses included in the criminal code, all drug-related offenses, and all ‘other’ criminal offenses |
| > 35 | ICD-8 | Register | No | All crimes: violent offenses, theft of all kinds, fraud, vandalism, traffic offenses included in the criminal code, all drug-related offenses, and all ‘other’ criminal offenses |
| 24-33 | NA | Self-report | Yes: adjust for sex, age, ethnicity, education, and social desirability response | Violent behaviour: recent fighting and weapon use in five-year period |
| 18-40 | DSM-III-R | Register, self-report,  and collateral  report | No | Violent behaviour: kick/bite/choke/hit/beat up and weapon use/weapon threat and acts that were coded as other aggressive acts were primarily throw objects/push/grab/shove |
| 22 | DSM-IV | Self-report  and parental  report | No | Violent acts: arson, assault resulting in injury to another person, breaking and entering, mugging, robbery, starting serious physical fights, threats to injure others, and vandalism |
| 16-74 | DSM-IV | Self-report | Yes: adjusted for sex, age social classes III-V, marital status, employment, any affective/anxiety disorder, alcohol dependence, drug dependence, and psychosis-positive screen | Violent behaviour: fight and weapon use |
| 16-74 | DSM-IV | Self-report | Yes: adjusted for sex, age social classes III-V, marital status, employment, any affective/anxiety disorder, alcohol dependence, drug dependence, and psychosis-positive screen | Violent behaviour: fight and weapon use |
| 18-23 | ICD-10 | Register | No | All crimes: drug, violent, property, traffic, and drunk driving offence |

*Information unavailable; ^a^Reflects *N* for both sexes combined; ^b^Reflects the presence of adjustment for the odds ratio included in our meta-analysis; ASPD = antisocial personality disorder; AvPD = avoidant personality disorder; BPD = borderline personality disorder; DPD = dependent personality disorder; HPD = histrionic personality disorder; NPD = narcissistic personality disorder; OCPD = obsessive compulsive personality disorder; PPD = paranoid personality disorder; ScPD = schizoid personality disorder; StPD = schizotypal personality disorder; GP = general population; *k* = number of studies

**Appendix Table B.2. Characteristics of Included Studies on the Risk of Recidivism in PDs**

| Study | Country | Study type | PDs (*N*) | Controls (*N*) | Cases | Comparison Group | Diagnostic criteria | Mean age | Sex | Definition of Outcome |
| --- | --- | --- | --- | --- | --- | --- | --- | --- | --- | --- |
| Additional studies identified in this updated review | | | | | | | | | | |
| Gray et al. (2011) | UK | Cohort | 160 | 730 | All PDs | Other psychiatric disorders | ICD-10 | 37.7 | Male | General and violent recidivism |
| Lund et al. (2012) | Sweden | Cohort | 55 | 91 | All PDs without comorbidity of other psychiatric disorders | Other psychiatric disorders without comorbidity | DSM-III  ICD-9 | 33 | Male | Recidivism |
| Dias et al. (2014) | Brazil | Cross-sectional | 105 | 503 | All PDs | Other psychiatric disorders | DSM-IV  ICD-10 | 17.3 | Mixed | Recidivism |
| Chang et al., F (2015) | Sweden | Cohort | 353 | 3133 | All PDs | Other psychiatric disorders | ICD-8 | * | Female | Violent recidivism |
| Chang et al., M (2015) | Sweden | Cohort | 2320 | 41520 | All PDs | Other psychiatric disorders | ICD-8 | * | Male | Violent recidivism |
| Krona et al. (2017) | Sweden | Cohort | 34 | 91 | All PDs | Other psychiatric disorders | * | 38 | Mixed | General and violent recidivism |
| Lim et al. (2017) | Korea | Cross-sectional | 14 | 106 | ASPD | Other psychiatric disorders | DSM-IV | 16.3 | Mixed | Recidivism |
| Seto et al. (2018) | Canada | Cohort | 677 | 814 | All PDs | Other psychiatric disorders | ICD-10 | 36.1 | Mixed | General and violent recidivism |
| Shepherd et al. (2018) | Australia | Cohort | 31 | 85 | ASPD | Other psychiatric disorders | DSM-IV | 32.2 | Mixed | General and violent recidivism |
| Forry et al. (2019) | Uganda | Cross-sectional | 86 | 328 | ASPD | Other psychiatric disorders | DSM-IV  ICD-10 | ≥18 | Mixed | Recidivism |
| Martin et al. (2019) | Spain | Cross-sectional | 50 | 50 | ASPD | No psychiatric disorders | DSM-5 | 40.3 | Male | Recidivism |
| Forget et al. (2022) | Canada | Cohort | 8 | 122 | All PDs | Other psychiatric disorders | * | 33.9 | Mixed | Recidivism |
| Klausing & Seifert (2022) | Germany | Cohort | 102 | 219 | All PDs | Other psychiatric disorders | DSM | * | Mixed | Recidivism |
| Mitchell et al. (2023) | Australia | Cohort | 305 | 1341 | All PDs | Other psychiatric disorders | ICD-10 | 38.1 | Mixed | Recidivism |
| Ogilvie et al. (2023) | Australia | Cohort | 459 | 23071 | All PDs | No psychiatric disorders | ICD-9  ICD-10 | 21.3 | Mixed | General and violent recidivism |
| Okamura et al. (2023) | Japan | Cohort | 8 | 140 | All PDs | Other psychiatric disorders | ICD-10 | 44.3 | Mixed | Recidivism |
| Yukhnenko et al., F (2023) | Sweden | Cohort | 1324 | 10448 | All PDs | Other psychiatric disorders | ICD-8  ICD-9  ICD-10 | 28 | Female | General and violent recidivism |
| Yukhnenko et al., M (2023) | Sweden | Cohort | 2671 | 67972 | All PDs | Other psychiatric disorders | ICD-8  ICD-9  ICD-10 | 28 | Male | General and violent recidivism |
| Capuzzi et al. (2024) | Italy | Cross-sectional | 62 | 417 | All PDs | Other psychiatric disorders | DSM-5 | 41.7 | Male | Recidivism |
| Cohen et al. (2024) | USA | Case-control | 220 | 174 | All PDs | Other psychiatric disorders | DSM-5 | 34.5 | Mixed | Recidivism |
| Studies identified in the 2012 review | | | | | | | | | | |
| Ganzer & Sarason, F (1973) | USA | Case-control | 60 | 40 | All PDs | Other psychiatric disorders | * | 14.7 | Female | Recidivism |
| Ganzer & Sarason, M (1973) | USA | Case-control | 70 | 30 | All PDs | Other psychiatric disorders | * | 15.3 | Male | Recidivism |
| Quinsey et al. (1975) | Canada | Cohort | 44 | 47 | All PDs | Other psychiatric disorders | * | 32 | Male | Recidivism |
| Martin et al. (1978) | USA | Cohort | 42 | 22 | ASPD | Other psychiatric disorders | * | * | Female | Recidivism |
| Tennent & Way (1984) | UK | Cohort | 226 | 262 | All PDs | Other psychiatric disorders | * | * | Male | Recidivism |
| Yesavage et al. (1986) | France | Cohort | 340 | 756 | All PDs | Other psychiatric disorders | DSM-III | * | Mixed | Recidivism |
| Rice et al., a (1990) | Canada | Cohort | 47 | 206 | All PDs | Other psychiatric disorders | DSM-III | 30 | Male | Recidivism |
| Rice et al., b (1990) | Canada | Cohort | 84 | 131 | All PDs | Other psychiatric disorders | DSM-III | 29.6 | Male | Recidivism |
| Komer & Galbraith (1992) | Canada | Cohort | 15 | 15 | All PDs | Other psychiatric disorders | * | 28.4 | Mixed | Recidivism |
| Bailey & Macculloch (1992) | UK | Cohort | 62 | 50 | ASPD | Other psychiatric disorders | DSM-III-R | * | Male | Recidivism |
| Harris et al. (1993) | Canada | Cohort | 225 | 393 | All PDs | Other psychiatric disorders | DSM-III | 27 | Male | Violent recidivism |
| Russo (1994) | Italy | Cohort | 22 | 70 | ASPD | Other psychiatric disorders | * | 40 | Male | Recidivism |
| Porporino & Motiuk (1995) | Canada | Cohort | 18 | 9 | ASPD | No psychiatric disorders | DSM-III | 33.6 | Male | Recidivism |
| Harris & Koepsell (1996) | USA | Cohort | 6 | 21 | All PDs | Other psychiatric disorders | * | * | Mixed | Recidivism |
| Ventura et al. (1998) | USA | Cohort | 94 | 167 | All PDs | Other psychiatric disorders | DSM-III-R | 28.7 | Mixed | General and violent recidivism |
| Singleton et al., F (1998) | UK | Cohort | 416 | 153 | All PDs | Other psychiatric disorders | DSM-IV | * | Female | Recidivism |
| Singleton et al., M (1998) | UK | Cohort | 810 | 296 | All PDs | Other psychiatric disorders | DSM-IV | * | Male | Recidivism |
| Monson et al. (2001) | USA | Cohort | 41 | 84 | ASPD | Other psychiatric disorders | * | 37.7 | Mixed | Recidivism |
| Moscatello (2001) | Brazil | Cross-sectional | 29 | 71 | All PDs | Other psychiatric disorders | ICD-10 | 38.9 | Male | Recidivism |
| Bertman-Pate et al. (2004) | USA | Cohort | 28 | 91 | All PDs | Other psychiatric disorders | DSM-IV | 38 | Mixed | Recidivism |
| Stadtland & Nedopil (2005) | Germany | Case-control | 28 | 75 | All PDs | Other psychiatric disorders | ICD-10 | * | Mixed | Recidivism |
| Coid et al., F (2007) | UK | Cohort | 56 | 112 | All PDs | Other psychiatric disorders | DSM-III-R | 31.6 | Female | Recidivism |
| Coid et al., M (2007) | UK | Cohort | 132 | 980 | All PDs | Other psychiatric disorders | DSM-III-R | 31.6 | Male | Recidivism |
| Grann et al. (2008) | Sweden | Cohort | 2159 | 159 | All PDs | No psychiatric disorders | DSM-III | 35.7 | Mixed | Violent recidivism |
| Vitacco et al. (2008) | USA | Cohort | 33 | 330 | ASPD | Other psychiatric disorders | DSM-IV | 41 | Mixed | Recidivism |

*Information unavailable; ^a^Reflects *N* for both sexes combined; ASPD = antisocial personality disorder; *k* = number of studies.

**Appendix B. Sensitivity Analyses for Studies Reporting Any Criminality Associated with PD**

**Appendix Table C.1. Odds ratios of any antisocial behaviour (including violence) in individuals with PD compared with the general population**

| **Type of PD** | ***k*** | **OR (95% CI)** | ***I^2^*** |
| --- | --- | --- | --- |
| **Any PD*** | 5 | 5.0 (4.6 to 5.4) | 58 |
| **ASPD** | 4 | 5.2 (3.4 to 8.1) | 97 |
| **BPD** | 2 | 2.8 (1.1 to 7.0) | 99 |
| **StPD** | 1 | 1.0 (0.8 to 1.2) | n/a |

*Results were from fixed-effects model; ASPD = antisocial personality disorder; BPD = borderline personality disorder; *k* = number of studies; StPD = schizotypal personality disorder

**Appendix Figure C.1. Risk estimate for any antisocial behaviour (including violence) in individuals diagnosed with all PDs compared with the general population.**

**Appendix Figure C.2. Risk estimate for any antisocial behaviour (including violence) in individuals diagnosed with ASPD compared with the general population.**

**Appendix Table C.2. Risk estimates for any antisocial behaviour (including violence) in all PDs by study characteristics**

| **Sample or Study Characteristics** | **Number of Studies** | **Number of Cases with PD**  **(Antisocial behaviour cases)** | **Random-effects**  **OR (95% CI)** |
| --- | --- | --- | --- |
| **Study period (n = 5)** | | | |
| Studies conducted before 1998 | 4 | 429 (216) | 6.2 (3.9 to 10.0) |
| Studies conducted in and after 1998 | 1 | 6342 (676) | 5.0 (4.6 to 5.4) |
| **Study region (n = 5)** | | | |
| USA | 2 | 85 (36) | 9.2 (5.6 to 15.0) |
| The rest of the world | 3 | 6686 (856) | 4.8 (4.0 to 5.9) |
| **Design (n = 5)** | | | |
| Cohort | 1 | 56 (14) | 9.9 (5.4 to 18.2) |
| Cross-sectional | 4 | 6715 (878) | 4.9 (4.1 to 6.0) |
| **Adjustment (n = 5)** | | | |
| With adjustment | 2 | 344 (180) | 4.8 (2.9 to 7.9) |
| Without adjustment | 3 | 6427 (712) | 6.7 (4.0 to 11.0) |
| **Comparison group (n = 5)** | | | |
| General population | 4 | 429 (216) | 6.2 (3.9 to 10.0) |
| General population without psychiatric disorders | 1 | 6342 (676) | 5.0 (4.6 to 5.4) |
| **Sex (n = 4)** | | | |
| Male | 3 | 288 (174) | 6.3 (3.2 to 12.5) |
| Female | 1 | 141 (42) | 6.4 (3.9 to 10.5) |
| **Number of cases (n = 5)** | | | |
| <100 cases | 2 | 85 (36) | 9.2 (5.6 to 15.0) |
| 100-1000 cases | 2 | 344 (180) | 4.8 (2.9 to 7.9) |
| >1000 cases | 1 | 6342 (676) | 5.0 (4.6 to 5.4) |
